# Failure rates and complications of four sphincter-sparing techniques for the treatment of fistula-in-ano: a systematic review and network meta-analysis

**DOI:** 10.1007/s10151-025-03152-0

**Published:** 2025-05-20

**Authors:** G. Fuschillo, F. Pata, M. D’Ambrosio, L. Selvaggi, M. Pescatori, F. Selvaggi, G. Pellino

**Affiliations:** 1 Colorectal Surgery, Vall d’Hebron University Hospital, Universitat Autonoma de Barcelona UAB, Barcelona, Spain; 2 Department of Advanced Medical and Surgical Sciences, Università degli Studi della Campania “Luigi Vanvitelli,” Policlinico CS, Piazza Miraglia 2, 80138 Naples, Italy; 3 Department of Pharmacy, Health and Nutritional Sciences, University of Calabria, Rende, Italy; 4 Department of Surgery, General Surgery Unit, A.O. Annunziata, Cosenza, Italy; 5 Coloproctology Units of Parioli and Cobellis Clinics, Rome and Vallo Della Lucania, Italy

**Keywords:** LIFT, VAAFT, FILAC, Endoanal flap, Anal fistula, Complications, Recurrence

## Abstract

**Background:**

Several techniques are available to reduce the risk of sphincter injury when treating anal fistula, such as ligation of the intersphincteric fistula tract (LIFT), video-assisted anal fistula treatment (VAAFT), fistula laser closure (FiLaC) and endoanal flap (EAF). The aim of this meta-analysis is to provide data on the safety, complications and failure of these techniques.

**Methods:**

Studies published after 2017, with patients undergoing at least one among LIFT, VAAFT, FiLaC and EAF for perianal fistula and providing data regarding failure, were retrieved from PubMed and EMBASE. Primary outcome was failure; other outcomes included continence disturbance and complications.

**Results:**

Forty-nine articles with 3520 patients were included. The failure rates were 28.6% (range 3.8–75) for LIFT, 22.3% (6.2–65.2) for VAAFT, 43.9% (11.1–80) for FiLaC and 25.9% (4.7–100) for EAF, with a mean follow-up of 35.4 (6–80.4), 32.4 (6–48), 31.6(6.3–60) and 42.4 (12–155) months. The available network meta-analysis on failure showed RD of –0.08 (95% CI − 0.58 to 0.42) comparing LIFT vs VAAFT and 0.30 (95% CI 0.03 to 0.58) comparing LIFT vs EAF. No patients undergoing VAAFT or FiLaC reported worsening continence, while for LIFT and EAF, the continence disturbance rate was 1.5% and 7.3%, respectively. No major complications were observed. The most common minor complications were pain (1.4%), bleeding (1.1%) and wound infection (1.2%). Overall, minor complication rates were 4.3% for LIFT, 7.2% for VAAFT, 10.2% for FiLaC and 6.2% for EAF. Crohn's disease was associated with a higher failure rate (39.5% vs 31.4%).

**Conclusions:**

FiLaC, VAAFT, LIFT and EAF may represent a valid option in the treatment of anal fistula. VAAFT showed the lowest rate of failure but with no differences from network metanalysis. Wider homogeneous studies with long-term follow-up are necessary to obtain more robust data.

**PROSPERO number:**

CRD42022375600.

**Supplementary Information:**

The online version contains supplementary material available at 10.1007/s10151-025-03152-0.

## Introduction

The treatment of anal fistula is a challenge for both surgeons and patients due to its anatomical difficulties, the impact on the patient’s life and potential injury to sphincter function, which can undermine the trusting relationship between patients and doctors. Several techniques are currently available for the treatment of anal fistula [1], although the gold standard is still controversial [2]. The treatment goal is to offer definitive resolution balanced with the risk of disturbed fecal continence [1]. Therefore, many sphincter-saving procedures have been proposed, but the use of some of these has rapidly declined because of poor outcomes in terms of mid-term recurrence [3–5].

New minimally invasive techniques and new devices were recently introduced to reduce the risk of sphincter injury are showing encouraging results. The ligation of intersphincteric fistula tract (LIFT) procedure, for example, consists of an incision at the intersphincteric space, identification and ligation of the intersphincteric tract [6]. Video-assisted anal fistula treatment (VAAFT), presented in 2011 by Meinero et al. [7], involves using a fistuloscope, which is introduced through the external orifice and allows visualization of the fistula tract, and closure by cyanoacrylate instillation. The fistula laser closure (FiLaC) technique utilizes a radial emitting laser fiber, allowing destruction of the epithelium of the fistula and simultaneous closure of the remaining tract by laser-induced narrowing [8].

The endoanal flap (EAF) procedure is performed by excising the internal opening, which is covered with a submucosal flap with or without muscle fibers of the internal sphincter [9].

The mid- and long-term outcomes of such procedures and the associated complications need to be further elucidated.

The aim of this study is to provide data on the safety profile, postoperative complications and recurrence of these four techniques by assessing the available literature on the topic.

## Methods

The systematic review and meta-analysis were performed following the Preferred Reporting Items for Systematic Reviews and Meta-analysis (PRISMA) Statement [10] and registered on PROSPERO (CRD42022375600).

### Search strategy and data sources

The literature search was conducted on PubMed and Embase database. Two screeners independently performed the literature screening (GF and MD). Data were extracted independently from included studies by the reviewers. The search terms used were: “ligation of intersphincteric fistula tract,” “video assisted anal fistula treatment,” “fistula laser closure,” “endoanal flap” and “endorectal flap.” Cross-reference search was used. The detailed search strategy is shown in Supplementary Table 1.

### Inclusion and exclusion criteria

Only studies with patients undergoing FiLaC, LIFT, VAAFT and EAF for surgical treatment of anal fistula that provided data regarding the primary endpoint, specifically failure, were included in the analysis. Studies with non-extractable data, with no full text available and published before 2017 and those written in languages other than English were excluded. For more than one study conducted at the same center, only the most recent one was included.

### Endpoints

The primary endpoint was failure of each technique, defined as permanence of the fistulous tract or recurrence at the last available follow-up (FU).

The secondary endpoints were:

- continence disturbances, defined as the worsening of the patient's permanent continence following the procedure
- failure in patients with perianal Crohn’s disease
- any major and minor complications

### Data of interest

Data of interest were: first author, year of publication, country, study design, number of patients, number of patients with Crohn’s Disease, type of fistula, complex fistula rate (conventionally defined as fistulas with > 30% involvement of the external sphincter, those anterior in female patients or those with secondary tracts), mean age, male rate, mean follow-up, healing definition, preoperative fistula assessment, technique details, previous fistula surgery, number of failures, incontinence, complications and type of complications.

### Statistical analysis

All collected data regarding the endpoints were reported in an Excel table showing the number of patients involved in the studies, number of failures, incontinence, complications (and type) and Crohn’s disease. A proportional meta-analysis was realized to assess the mean rate of failure across the studies using MedCalc® statistical software. A network meta-analysis was performed to compare the four techniques using STATA software and Risk Difference (RD) as effect size. Sensitivity analyses were attempted for subgroups of patients if data allowed for it. Statistical heterogeneity was assessed using the *P* value of the Cochrane *Q* test and inconsistency (*I*^2^) statistics.

### Risk of bias assessment and quality of the studies

The Joanna Briggs Institute’s (JBI) critical appraisal checklist for studies reporting prevalence data was utilized to evaluate the quality of the studies [11]. This tool assessed studies according to ten questions. If the answer was yes, the question was assigned a score of 1. If the answer was no, unclear or not applicable, it was assigned a score of 0.

Data assessment was independently performed by two reviewers (GF and MD). Any disagreement was resolved by discussion with a third reviewer (GP).

As there is no clear consensus about their use in the context of a proportional meta-analysis [12], a funnel plot was not performed to assess the publication bias.

## Results

The search yielded 525 studies, and 32 duplicates were excluded. The titles of 493 articles were analyzed; abstracts, reviews, meta-analyses and case reports were excluded. Seventy-two full text articles were assessed for eligibility, of which three items were excluded because of lack of data regarding the endpoints and seven because they were conducted at the same center.

**Fig. 1:**
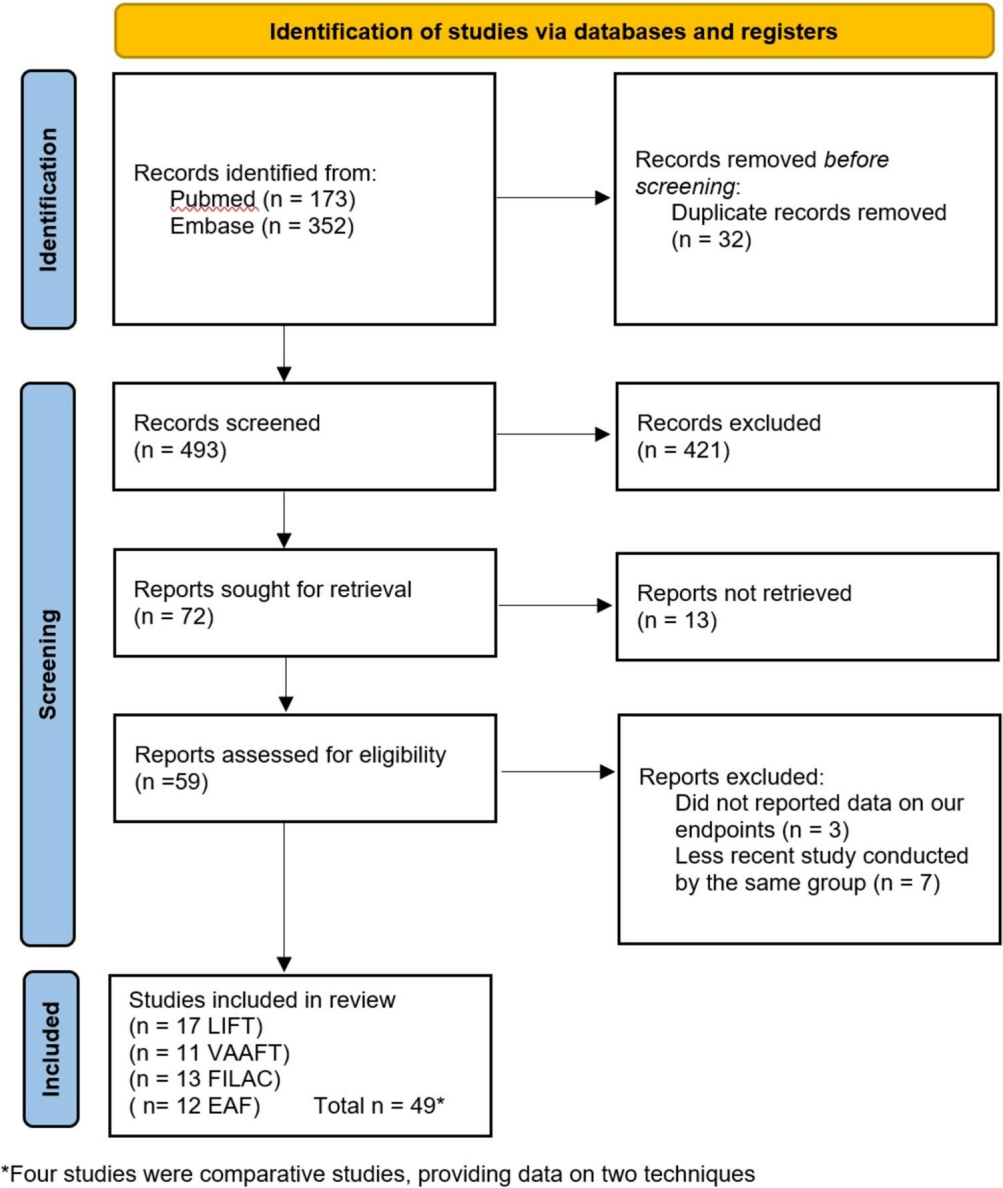
Flow chart of study selection according to the PRISMA statement

Forty-nine articles were included in the review, 17 concerning LIFT [13–29], 11 on VAAFT, [26, 30–39], 13 on FiLaC [40–52] and 12 on EAF [24, 28, 29, 53–61]. Four studies were comparative studies, providing data on more than one technique [24, 26, 28, 29]. The study inclusion flowchart is reported in Fig. 1.

There was 100% agreement among reviewers regarding data extraction. In total, 3540 patients were included in the analysis: 1068 patients underwent LIFT, 773 VAAFT, 851 FiLaC and 848 EAF. The collected data are summarized in Table 1 for VAAFT, Table 2 for LIFT, Table 3 for FiLaC and Table 4 for EAF.

**Table 1:** Characteristics of included studies (VAAFT)

First author	Years	Country	Study design	N PTS	CD	Fistula type	Complex rate	Mean age (years)	Male rate	Mean FU (months)	Previous fistula surgery	Healing definition	Assessment	Technical notes
La Torre et al.	2020	Italy	Retrospective	28	–	100% transphincteric	–	–	–	18	10.7%	Scarring of the intersphincteric wound and the external opening	EAuS and MRI	Surgical techniques were performed following all the steps described by Meinero et al. in 2011. No loose seton was placed before the procedure
Regusci et al.	2020	Switzerland	Prospective	96	0	77.9% high transphincteric 16.4% extrasphincteric 3.8% suprasphincteric 1.9% intersphincteric	100%	48.9	72.1%	36	24%	absence of fistula at clinical examination	Physical examination and ultrasonography in all patients. Colonoscopy, MRI and CT when required	VAAFT has two phases, a diagnostic one and an operative one, as previously described by Meinero et al. in 2011.Closure of the internal opening was performed using a mucosal flap, stapler device or application of absorbable sutures
Khan et al.	2022	Pakistan	Retrospective	76	0	–	–	35	97.6%	12	7.25%	No evidence of ongoing sepsis or discharge and closed internal and external openings on clinical examination	Digital rectal examination and proctoscopy. Patients suspected of complex fistulae were assessed with MRI scan	Fulguration with monopolar diathermy of the fistula tract followed by brushing of the tract to remove debris using an endo-brush and closure of internal opening using absorbable suture material (Vicryl 1)
Sørensen et al.	2021	Denmark	Randomized	23	0	100% high transphincteric	100%	42.6	74%	6	–	An epithelialized wound	Anal examination under general anesthesia, loose seton suture positioning, endoanal ultrasonography. MRI and colonoscopy when indicated	–
Giarratano et al.	2020	Italy	Prospective	72	–	86% transphincteric 13% extrasphincteric 1% suprasphincteric	100%	46	54%	32	19%	Complete closure of all external openings during the first 90 days postoperatively	Clinical examination and EAUS. Patients aged > 50 with a personal or family history of intestinal polyps or cancer were offered colonoscopy	Diagnostic phase identifies the fistula anatomy, any associated tracks and/or any abscess cavities. During this phase, the fistuloscope is introduced through the external opening passing through the fistulous track until it reaches the internal opening, which is identified and sutured with a 3–0 Vicryl® In the operative phase, an electrode is introduced through the operating channel, and the fistulous track and any associated cavities are ablated . It is then removed by a brush inserted through the operating channel or by using a Volkmann spoon. The continuous jet of the washing solution also ensures the full elimination of this material
Lopez et al.	2020	Philippines	Retrospective	16	0	45% high transphinteric, 30% low transphinteric, 20% suprasphincteric 5% intersphincteric	48.6%	38.2	90%	6	65%	Absence of perianal discharge and complete epithelialization of the perianal wound/external opening	–	The fistuloscope was passed through the external opening while filled with a saline-mannitol solution. The internal opening was confirmed by advancing the tip of the scope. Two or three sutures were placed at opposite points of the margin of the internal opening to isolate but not close this opening An electrode was used to electrodessicate the fistula tract under direct visualization. After thorough cleaning of the fistula tract, the internal opening was closed
Siddique et al.	2022	Pakistan	Randomized	40	0	50% transphincteric 35% intersphincteric 12.5% suprasphincteric 2.5% extrasphincteric	–	39.9	82.5%	36	O%	Complete closure of the external opening (perianal wound) without any complaints of discharge or pus from the scar	–	The external opening was widened with a probe, and a fistulascope was inserted to delineate the primary and secondary tracts and locate the internal opening. The internal opening was then stitched with Vicryl™ 2–0 suture through the anal route using a proctoscope. The fistula tract was washed and debrided using the scope and cauterized. Finally, the external opening was excised and sent for biopsy
Romaniszyn et al.	2017	Poland	Retrospective	68	0	–	71%	43.8	70.6%	31	–	–	–	The fistuloscope was introduced into the external opening, and the procedure was performed according to the description of Meinero and Mori in 2011, except for the closure of the internal opening, which was performed with either a “figure eight” suture (65 patients) or an advancement flap (3 patients) rather than using a stapler. The tracts were destroyed using electrocautery, the necrotic tissues removed and the external openings cored out and left open for drainage
Zelić et al.	2020	Croatia	Prospective	73	–	–	100%	45	65.7%	24	26%	Absence of discharge and closure of the external opening until 6 months postoperatively	Clinical examination, digital rectal examination, linear metal probe and MRI	The aim of the diagnostic phase was to visualize the entire fistula tract and the internal opening and to identify any possible secondary tracts and abscess cavities. Where the internal opening was covered by healthy mucosa, no further action was taken to avoid forming a false tract. In the therapeutic phase, complete destruction of the main and secondary fistula tracts and closure of internal opening are performed. We used mattress suture, rectal advancement flap (RAF) or ligation of intersphincteric fistula tract (LIFT) technique to close the internal opening
Stazi et al.	2018	Italy	Retrospective	224	0	67% transphincteric 15.2% horseshoe 7.1% suprasphincteric 6.3% extrasphincteric 4.5% intersphincteric	100%	43	69.6%	48	76,8%	–	–	–
Zhang et al.	2021	China	Retrospective	57	–	57.9% suprasphincteric 42.1% transhpincteric 28.1% horseshoe	100%	37.5	82.5%	28	42.1%	Absence of discharge and closure of the external opening for 6 months postoperatively	–	The fistuloscope was inserted through the external opening. If the tract was straight, we achieved good visualization of the lumen to locate and mark the internal opening by constant injection of glycine-mannitol 1% solution. The filler was removed, and the electrode was replaced, followed by continuous destruction of the fistula wall under direct vision. The fistula tract wall was slowly cauterized into white fragments along the fistula wall; the fistula wall was scratched by a brush until fresh tissue was visible. Then, the AFP was placed from the internal opening to the external opening to fill the tract, and both sides of the plug were sutured and fixed with 2–0 Polysorb™

**Table 2:** Characteristics of included studies (LIFT)

First author	Years	Country	Study design	N PTS	CD	Fistula type	Complex rate	Mean age (years)	Male rate	Mean FU (months)	Previous fistula surgery	Healing definition	Assessment	Technical notes
Malakorn et al.	2017	Thailand	Retrospective	251	–	89.3% transphincteric 10.7% intersphincteric	–	41	82.5%	71	16.7%	Absence of symptoms with no visible external opening on clinical examination	Physical examination	Details of the technique are identical to those originally proposed in 2007 by Rojanasakul et al.
Kaminski et al.	2017	USA	Retrospective	23	23	100% transphincteric	100%	39	47.8%	23	26.1%	Complete healing of the surgical intersphicteric wound and closure of external opening without anal pain	Physical examination	–
Xu et al.	2017	China	Retrospective	55	6	56.4% transphincteric 36.4% intersphincteric 7.2% rectovaginal	100%	46	63.6%	16	100%	No evidence of residual fistula tract, closure of the internal and opening	EAUS	Using blunt dissection in the intersphincteric plane, the internal and external sphincter muscles were separated to expose the fistula tract. Once the tract is dissected free, it is encircled, and the probe can be removed. Next, the fistula tract is divided and ligated. The incision was closed with absorbable sutures after the wound was irrigated. The external opening was left open to drain
Kang et al.	2018	Korea	Prospective	28	0	100% transphincteric	32%	44	74%	16	21%	Complete epithelization of the external wound without any discharge or discomfort	Physical examination and EAUS	An encircling incision was made around the external fistula opening, and dissection was started from the external opening, along the fistula tract, toward the internal opening. Meticulous dissection was performed around the fistula tract so as not to injure the sphincter muscle, and this was continued until the distal part of the internal sphincter was separated from the fistula tract. When the dissection was finished, a mosquito clamp was applied to the separated fistula tract as proximally as possible. The fistula tract was then doubly ligated with Vicryl 2–0 or 3–0 at the proximal part of the clamp. The distal part of the ligated fistula tract was resected using Metzenbaum scissors. A cored-out wound was left open for drainage
Wen et al.	2018	China	Retrospective	62	0	83.9% transphincteric 16.1% intersphincteric	72.6%	34	69.4%	24.5	29%	Complete healing of the surgical intersphincteric wound and the external opening without any sign of recurrence	MRI	A1.5–2.0-cm curved incision was made at the anal canal skin to disconnect the intersphincteric fistula between the internal and external sphincters before it reached the intersphincteric groove; the intersphincteric fistula was lifted with right angle forceps. Purse-string suture around the fistula was then introduced for the ligation of the fistula at the side of internal sphincter with 3/0 Vicryl close to the lateral side of internal anal sphincter. Similarly, purse-string suture around the fistula was performed to ligate the fistula at the side of external sphincter with 3/0 Vicryl close to the interior side of external anal sphincter. Next, the residual fistulas were removed in a tunnel-based way to the external sphincter border. The external opening was unclosed for drainage. Intersphincteric was sutured with 4/0 Vicryl, perianal skin and the subcutaneous arc-shaped incisions were sutured with 3/0 Vicryl
Vander Mijnsbrugge et al.	2019	The etherlands	Prospective	45	0	96% transphincteric 4% ano-introital	91%	40	37.8%	12	71%	–	EAUS and Physical examination	Essential steps of the procedure include incision at the intersphincteric groove, identifcation of the intersphincteric portion of the tract, thorough cleaning of the tract, ligation of intersphincteric tract close to the internal opening, removal of intersphincteric portion of the tract, coring out of the external tract and external opening, and suturing of the defect at the intersphincteric site of the external sphincter muscle; the external opening was left open for discharge
Gottgens et al.	2019	The Netherlands	Retrospective	42	0	100% transphincteric	–	42.5	59%	6	13%	External opening is completely closed, without signs of septic criteria/signs of abscess	MRI 98%; EAUS 2%	The seton is removed, and a metal probe is inserted through the fistula tract. A circular incision is made in the intersphincteric groove, and a self-retaining retractor is installed for traction. With diathermia, the intersphincteric plane is developed until the intersphincteric fistula tract appears. The fistula is circled with a clamp until there is enough space to ligate it. The probe is removed, and the external and internal sides of the fistula are transfixed with Vicryl 2–0. The wound is then approximated with fast-resorbable sutures but not closed completely
Zhao et al.	2019	China	Retrospective	78	0	100% transphincteric	100%	43	42.1%	30	0%	No symptoms and signs of fistula, closing of internal and external opening, closing of intersphincteric groove wound	Digital rectal examination, proctoscopy and EAUS	The external opening was identified and enlarged for drainage. Then, a metal fistula probe was inserted into the fistula tract. A 1.5-cm to 2.0-cm curvilinear incision was made along the inter-sphincteric groove above the fistula tract. A separate fistula was formed between internal and external sphincters, the fistula was cut as close as possible to the internal sphincter, and the fistula opening was sutured at the internal sphincter with a 3/0 absorbable suture. The fistula was retained from the external sphincter to the external opening. The infected granulation tissue was gently scraped away and washed with metronidazole saline. A 3 × 5-cm sheet of human acellular dermal matrix was introduced from the external opening into the fistula tract and was sutured and fixed with 3/0 absorbable sutures at the external sphincter. The wound was loosely sutured intermittently with 3/0 absorbable sutures
Mujukian et al.	2020	USA	Retrospective	38	38	68.4% transphincteric 29% anovaginal 2.6% pouch perianal	100%	35	42%	27	34%	Closure of all external wounds and cessation of drainage and pain	MRI	–
Tsang et al.	2020	Hong Kong	Retrospective	58	0	86.2% transphincteric 12.1% extrasphincteric 1.7% suprasphincteric	100%	48	81%	15.25	86.2%	Complete healing of the external opening as well as the perianal skin incision	–	The fistula was cannulated using Lockhart-Mummery probes. A curvilinear skin incision was made over the intersphincteric groove. A combination of sharp and blunt dissection with S-shaped retractors was used to isolate the fistula tract. Suture ligation of the fistula tract was performed at proximal and distal ends using 4–0 Vicryl sutures. The tract was divided with or without excision of a segment of the fistula tract. The muscle defect medial to the external anal sphincter was sutured and approximated. The perianal incision was closed with interrupted 4–0 Vicryl sutures to complete the procedure
Chen Lau et al.	2020	Australia	Retrospective	116	0	100% transphincteric	100%	38	64.7%	36.4	57.8%	Closure of external opening and absence of clinical symptoms and discontinuity of fistula tract	EAUS and Manometry	A curvilinear skin incision was made over the intersphincteric groove. Using a Lone Star Retractor System™, the intersphincteric plane was dissected out and the intersphincteric portion of the fistulous tract was identified using right-angled forceps. The tract was suture ligated with either 2–0 dissolvable monofilament or braided suture and divided without excision. The external opening was excised and fistulous tract was curetted to the level of the external sphincter. The internal opening of the fistula was left alone. The surgical wound was then closed with dissolvable 3–0 dissolvable braided interrupted sutures
Van Praag et al.	2020	The Netherlands	Retrospective	19	19	–	–	34.5	31.6%	10.1	31.6%	Closure of external opening without discharge of pus or stools	MRI 94.7%	The intersphincteric plane was diathermically opened. Thereafter, the fistula tract was dissected and ligated close to the internal sphincter with an absorbable suture. The external fistula opening and subcutaneous tract were then excised and/or curetted. The intersphincteric wound was closed with absorbable sutures.
Zwiep et al.	2020	Canada	Retrospective	119	0	100% transphincteric	56.3%	45	56.9%	21.6	0%	Complete healing of both the external opening and intersphincteric incision	Physical examination and MRI	Draining setons were placed before repair The draining seton was removed and replaced with a fistula probe. The intersphincteric groove was identified, and an incision was made over it. A careful dissection in the intersphincteric plane was performed, and the fistula tract was isolated. After removal of the fistula probe, the tract was ligated proximally and distally and then divided. The external opening was enlarged and the tract curetted out. The skin was then reapproximated. The external opening was left open. The BioLIFT technique followed the same steps for identification, division and ligation of the fistula tract. After ensuring that both sides of the tract were ligated, a small piece of 7 × 10–cm Biodesign mesh trimmed to size was parachuted down into the wound to cover and separate the two ends of the tract
La Torre et al.	2020	Italy	Retrospective	26	-	100% high transsphincteric	–	–	–	18	11.5%		EAuS and MRI	The surgical techniques were performed following all the steps described by Rojanasakul et al., although no loose seton was placed before the procedure. The authors performed ligation of the immature intersphincteric tract with the surrounding dense scar tissue caused by inflammatory absorption
Wood et al.	2022	USA	Prospective	46	46	76% transphincteric 22% Anovaginal 2% Pouch-perineal	100%	34.2	40%	33.1	27%	Complete healing of the surgical inter-sphincteric wound and closure of the external opening without anal pain	–	–
Kumar et al.	2023	India	Randomized	42	0	–	–	–	–	24	–	–	–	–
Khan et al.	2024	USA	Retrospective	20	0	5% intersphincteric 15% Low transphincteric 80% Mid/high transphincteric	80%	–	–	80,4	–	–	–	–

**Table 3:** Characteristics of included studies (FILAC)

First author	Years	Country	Study design	N PTS	CD	Fistula type	Complex rate	Mean age (years)	Male rate	Mean FU (months)	Previous fistula surgery	Healing definition	Assessment	Technical notes
Wilhelm et al.	2017	Germany	Retrospective	117	13	76.9% transphincteric 11.1% supersphincteric 6.8% intersphincteric 5.1% extrasphincteric	–	46	70%	25.4	13.7%	Primary success	Clinical examination, proctosigmoidoscopy and 3D EAUS	The external and internal orifices of the fistula track were excised, followed by the preparation of a flap. Depending upon the local tissue situation in the area of the internal opening either an advancement, mucosal or anodermal flap was made. The fistula track was cleaned mechanically using a curette and irrigated with saline. The internal opening within the internal sphincter muscle was closed using a 2/0 Vicryl suture. The laser probe was inserted through the perineal fistula opening. This type of laser delivers energy at a wavelength of 1470 nm providing an optimal absorption curve in water, which is considered to result in a more efficient local tissue shrinkage and protein denaturation. For obliteration, the fistula track is treated with a continuous slow retraction of the laser fiber withdrawn at a rate of approximately 1 cm per 3 s
Lauretta et al.	2018	Italy	Retrospective	30	–	–	–	40	48.5%	11.3	73.3%	Complete healing of the surgical wound and external opening for at least 6 months	Clinical examination, anoscopy, 3D EAUS, peroxide hydrogen instillation. MRI was not routinely performed	The procedure was performed using the technique described by Giamundo et al. A diode laser platform emitting laser energy of 12 W at a wavelength of 1470 nm was used. The probes emitting laser energy radially have a diameter of 600 microns and are disposable. No particular treatment was used for the internal orifice
Marref et al.	2019	France	Prospective	68	6	66% high transphincteric 16% suprasphincteric 15% low transphincteric 3% intersphincteric	–	40	48.5%	6.3	54.4%	–	–	the seton was removed, the track was cleaned mechanically using a curette; then, the 1470-nm-diameter laser fiber delivered 12–15 W power from the internal to the external orifice. The internal orifice was not closed surgically
Isik et al.	2020	Turkey	Retrospective	100	–	54% high transphincteric 28% low transphincteric 10% intersphincteric 8% suprasphincteric	–	42	72%	48	–	No discharge, no symptoms, fibrotic scar on skin where previously external opening was present	MRI	The authors performed curettage of the fistula tract by using a plastic cytology brush. The laser probe was then inserted into the fistula tract via an external opening until the tip of the probe was located 1 or 2 mm beyond the internal opening. Energy was then applied as the laser probe was withdrawn through the external opening at a speed of 1 cm/6 s. During the application of the energy, letting the laser probe pass spontaneously through the fistula tract as it sealed the tract yielded the desired withdrawal speed. No sutures were placed at the internal or external openings, and no dressings or topical medications were used
Serin et al.	2020	Turkey	Retrospective	35	–	60% intersphincteric 34% transphincteric 6% supra/extrasphincteric	40%	43.9	68.5%	11		–	Clinical examination, rigid rectoscopy and/or flexible sigmoidoscopy and MRI (in patients who had a complex and/or recurrent fistula)	The laser fiber was introduced into the fistula tract via the external orifice. The fiber delivered laser energy homogeneously at 3600, causing shrinkage of the fistula tract around the fiber while it was withdrawn at the speed of 1 mm/s.2 At the end of each FiLaC procedure, the external and internal orifices of the fistula tract were debrided. An additional surgical technique was made as the closure of the internal orifice with a purse-string suture using 2–0 polyglactin. The external orifice was left open after curettage
Giamundo et al.	2021	Italy	Retrospective	175	0	86.8% transphincteric 10.3% intersphincteric 2.9% suprasphincteric	–	43.9	66%	60	85.7%	Closure of the external opening and absence of pain and discharge from both the external and internal orifices	Physical examination, proctoscopy and EAUS. MRI in patients with multiple orifices or those with repeatedly recurring fistulas	A 1000-µm-diameter radial laser probe (FiLaC®) was introduced into the fistula tract through the external orifice. The probe is powered by a diode laser platform and set at a wavelength of 1470 nm. Once the tip of the probe had reached the internal opening in the anal canal, the laser was activated and 12-W laser energy delivered in a continuous mode within the lumen of the tract while the probe was withdrawn at a constant speed of 1 mm/s. The procedure was deemed completed once the probe was completely extracted from the fistula tract. Closure of the internal opening was not routinely performed
Stijns et al.	2019	The Netherlands	Retrospective	20	0	70% transphincteric 30% intersphincteric	–	45	20%	10	10%	A fistula was considered healed if the external opening was completely closed without signs of sepsis or abscess	MRI	The external opening was probed gently to identify the fistula tract and internal opening. We used 2/0 Vicryl© as a guide wire placed through this tract. The CORONA™ fistula probe was used with a wavelength of 1470 nm. A laser energy of 10 W was used to seal the tract. The probe was withdrawn incrementally at a speed of about 1 mm/s to seal the tract, starting at the internal opening. No additional treatment (flap or suture) was used to close the internal opening. The authors did not perform any dissection or curettage of the external part of the fistula tract
Wolicki et al.	2020	Germany	Retrospective	83	2	–	–	50	77%	42	91.6%	Closure of the internal orifice and external orifice and the absence of pain or leakage	Digital examination, procto-sigmoidoscopy and EAUS. If necessary, MRI	The laser fiber was introduced through the external orifice in Seldinger technique using the seton as a guidewire. After it was placed on the mucosal level, the fiber was withdrawn with a speed of 1 mm per 1 s by applying 12 W energy. Therefore, the energy delivered per centimeter is 120 J. The internal orifice was closed using a 3/0 Vicryl suture and performing a Z-stitch. The external orifice was excised with a diameter of 10–15 mm and sent for histological examination
Brabender et al.	2020	USA	Retrospective	18	4	66.7% transphincteric 23.8% intersphincteric 4.8% suprasphincteric 4.8% superficial	–	41	56%	29	83.3%	No sign of leakage and complete resolution of symptoms	Anorectal exam and exam under anesthesia. MRI or EAUS was not used routinely	The tract was irrigated with normal saline. The laser probe was then placed within the fistula tract via the external opening. The laser was then fired in bursts as the fiber was slowly withdrawn through the fistula tract from the internal opening to the external opening. For the procedure, a laser fiber with an average of 10.63 + 1.3 W at a wavelength of 1470 nm was used. The internal opening was also suture ligated with 2–0 Vicryl suture and the external opening enlarged
Alam et al.	2019	France	Retrospective	20	20	70% high transphincteric 15% low transphincteric 5% intersphincteric 5% suprasphincteric 5% extrasphincteric	–	32	50%	7	–	When the internal and external openings were closed and the patient experienced no pain or leakage (spontaneously or under pressure)	–	The seton was removed, the track was debrided by rubbing, and then, the 1470-nm-diameter laser fiber delivered 13 W of power from the internal orifice to the external orifice
Nordholm-Carstensen et al.	2021	Denmark	Retrospective	66	11	60% high transsphincteric 29% low transsphincteric 7% suprasphincteric 3% high intersphincteric	–	40	42%	19	32%	No evidence of fluid-containing tracts or abscess on MRI OR no air-containing tracts on EAUS AND a healed external opening	MRI and EAUS	Briefly, the inner opening was closed within the internal sphincter with Polysorb ™ 2–0 suture. The fistula tract was not curettaged. In all cases, the diode laser probe was introduced from the outer opening and advanced to and through the internal opening. A wavelength of 1470 nm and power of 13 W were used resulting in controlled 2–3-mm-deep tissue damage, which minimizes the risk of perifistular thermal damage and should ensure the optimal depth for destruction of granulation tissue and fistula epithelium. The ablation began at the point of the internal opening and proceeded with continuous retraction aiming for 6 s of ablation per cm fistula track. Finally, the sutures over the internal opening were tied and the external opening excised or curettaged to ensure proper drainage of debris
Bonnechose et al.	2020	France	Retrospective	92	0	79% high transsphincteric 13% suprasphincteric 8% low transsphincteric	100%	43	65%	13.6	–	When the internal and external openings were closed and the patient experienced no pain or leakage	MRI and/or EAUS was not routinely used	After removing the seton, the tract was debrided with a specific brush. Treatment was performed using a 1470-nm-wavelength laser probe and standardized 13 W power in continuous mode. During the procedure, the internal orifice was not closed. The size of the internal orifice was estimated by the operator: it was considered narrow when its diameter was equal to that of the laser probe and wide when it was larger than the probe
Donmez et al.	2017	Turkey	Retrospective	27	–	51.8% intersphincteric 26% transsphincteric 18.5% suprasphincteric 3.7% extrasphincteric	–	35,5	85%	22	–	–	clinical examination, proctosigmoidoscopy and MRI	The fistula tract was mechanically cleaned using a curette and washed with saline. The laser probe was inserted into the external opening and passed through the internal opening. During application, the laser probe was allowed to pass through the fistula tract by itself and was manually withdrawn when its path was obstructed. Gently withdrawing the probe a few centimeters and then advancing it back toward the internal opening was sufficient to eliminate any untreated sections of fistula tract. The internal and external openings were not sutured

**Table 4:** Characteristics of included studies (EAF)

First author	Years	Country	Study design	N PTS	CD	Fistula type	Complex rate	Mean age (years)	Male rate	Mean FU (months)	Previous fistula surgery	Healing definition	Assessment	Technical notes
Kumar et al.	2023	India	Randomized	42	0	–	–	–	–	24	–	–	–	–
Khan et al.	2024	USA	Retrospective	1	0	100% intersphincteric	0%	50	63.5	80.4	–	–	–	–
Van Praag et al.	2020	The Netherlands	Retrospective	19	19	–		33.3	42.9%	40.9	14,3%	Closure of external opening without discharge of pus or stools	MRI 94.7%	The internal opening was excised and the fistula tract curetted. A (sub)mucosal flap (with or without muscle fibers of the internal sphincter) with vital edges was mobilized, after which the internal opening was closed with absorbable sutures (Vicryl 2/0). The internal fistula opening was then covered with the flap of (sub)mucosal tissue and sutured in the distal rectal technique
Jafarzadeh et al.	2019	Iran	Randomized	21	0	–	–	–	61.9%	12	–	Complete epithelialization of the wound	Imaging and endoscopy	The internal tract was used to enter the appropriate probe without any resistance through the outer hole of the fistula into the tract as much as possible. Subsequently, the epinephrine solution was injected at a ratio of 1:200,000 to the area under the mucosa and flap. Then, the V-shaped flap was removed in the mucosa and submucosa of the rectum at the site of the internal orifice. By removing the area with the hole or inner orifice, the inner hole at the muscle surface was closed using PDS 3–0 thread. Using flap advancement through the inner orifice, an external tract fistulectomy was performed to the extent of the muscles
Egal et al.	2020	France	Retrospective	35	5	77.1% Anovaginal 22.9% Anterior perineal	–	39	–	31	28.6%	Fistula closure at the proctological follow-up examination together with complete resolution of the passage of flatus or stool per vaginam	–	An anterior transverse incision was made distal to the internal opening extending to the submucosa, and a U-shaped flap consisting of mucosa and submucosa was prepared. The distal part of the flap, including the internal opening, was excised. The internal opening was closed with a figure 8 stitch, and then a transverse plication of the muscular layer and the internal anal sphincter and/or rectal muscular layers, depending on the height of the fistula, was made using an overthread or interrupted sutures. Finally, the mucosal-submucosal flap was advanced to cover the muscular plication and closed without tension by 3 sutures distally and 1 suture laterally
Bessi et al.	2019	France	Retrospective	87	34	27.6% infraelevator 72.4% supraelevator	89.8%	43.7	40.2%	13.3	–	–	–	The external opening was enlarged and the fistulous tract was excised as far as possible without reaching the external sphincter. The internal opening of the fistula was excised. Two to three stitches provided a plication of the muscle, facing and occluding the internal opening. Then, the submucosal area was dissected to create a flap with a buttonhole shape. The plicated muscle was covered with the mucosal flap thus produced. All stitches were performed with dissolvable sutures
Boenicke et al.	2017	Germany	Prospective	61	0	74% transphincteric 26% suprasphincteric	100%	45	73%	25	11%	Complete wound healing and absence of pain, bleeding or secretion and inconspicuous transanal ultrasound findings at 6-month follow-up	Transanal ultrasound and intraoperative findings	The seton was removed, and the fistula tract was dissected from the external opening to the intersphincteric space. The intrasphincteric part of the fistula was eliminated by curettage. The internal opening including the anoderm and the crypt-bearing tissue was excised. After irrigation, the defect in the internal sphincter was closed with a layer of interrupted PDS 2/0 sutures. A full-thickness rectal wall flap was raised from the level of the dentate line and mobilized over a distance of 3 cm. Overlapping the internal opening tension-free, it was fixated using Vicryl 3/0. The external opening was not closed to allow drainage
Bondi et al.	2017	Norway	Randomized	46	0	100% transphincteric	0%	42.2	45.8%	12	28.3%	Total absence of secretion, a dry scar at the external fistula opening and the absence of deep infection or cavities	MRI	Advancement flap surgery was performed by careful excision of the fistula tract from the internal to the external opening, leaving the external fistula opening to heal from the inside outwards. The internal fistula opening was covered directly by a mobilized full-thickness musculomucosal flap with appropriate blood supply and with a broad base involving between one quarter and one half of the rectal circumference. The flap was pulled in the caudal direction to cover the internal fistula opening with sufficient side margins but without tissue tension. The flap was fixed with absorbable sutures
Chaveli Diaz et al.	2021	Spain	Retrospective	115	–	88.5% Transphincteric 10% Suprasphincteric 1.5% Extrasphincteric	–	48.9	77.4%	155.5	73.8%	–	Digital examination, proctosigmoidoscopy, and rectal endosonography	The main fistulous tract and any secondary tracts were dissected, a small elliptical incision around the internal opening was made, and the fistula tract was excised. The internal opening was sutured with interrupted 4/0 polyglactin sutures. A curvilinear (semicircular) endorectal advancement flap of partial thickness was raised immediately above the internal opening and mobilized 4 ± 6 cm proximally. The base of the flap was approximately twice the width of its apex. The flap was advanced and sutured to the level of the intersphincteric line. The external wound was left open for drainage and packed loosely
Seifarth et al.	2021	Germany	Retrospective	155	55	83% transphincteric 15% rectovaginal 1% suprasphincteric 1% intersphincteric	–	40	54%	15.7	–	Complete healing of the fistulous tract (clinically and in anal endoscopic ultrasound) without the need for reoperation or replacement of the seton drain	–	The internal fistula opening is closed with a fap including the lamina muscularis and the mucosa of the rectum wall. The external opening of the fistula is excised or debrided
Uribe et al.	2020	Spain	Retrospective	190	0	83.7% transphincteric 16.3% suprasphincteric	–	50	70.5%	44.6	16.9%	Cessation of drainage and closure of external openings	–	After performing a coring out or curettage of the fistulous tract, a full-thickness flap consisting of mucosa, submucosa and the internal anal sphincter was created, advanced over the sutured internal opening and fixed with absorbable tension-free sutures
Yellinek et al.	2019	USA	Retrospective	76	–	–	100%	45.5	63.1%	13.8	46%	–	–	A full-thickness flap consisting of mucosa, submucosa and some of the circular muscle fibers was mobilized from the level of the dentate line to 4–5 cm cephalad. The base of the flap was at least twice as wide as its apex to ensure an adequate blood supply to the distal end. The distal part of the flap that contained the internal opening was excised, the fistulous tract was curetted and the internal portion of the fistulous tract was closed with absorbable sutures. The edge of the flap was advanced onto the dentate line and sutured without tension over the internal opening, using absorbable sutures. In cases of peri-anal fistula, the external opening was debrided and left open to drain or, according to the surgeon’s choice, was drained using a mushroom catheter secured in the fistulous tract. We do not typically use the ‘core out’ technique as it has shown higher rates of incontinence

Most of the included studies were retrospective. The nature of the included study differed according to the technique: for LIFT, 13 studies were retrospective, 3 prospective and 1 randomized (LIFT vs. EAF [28]); for VAAFT, 6 were retrospective, 3 prospective and 2 randomized (one comparing VAAFT vs fistulectomy [32] and one VAAFT vs seton [35]); for FiLaC, 12 were retrospective and 1 prospective; for EAF, 8 were retrospective, 1 prospective and 3 randomized (comparing EAF with LIFT [28], seton [53] and collagen plug [57], respectively).

### Failure

**Fig. 2:**
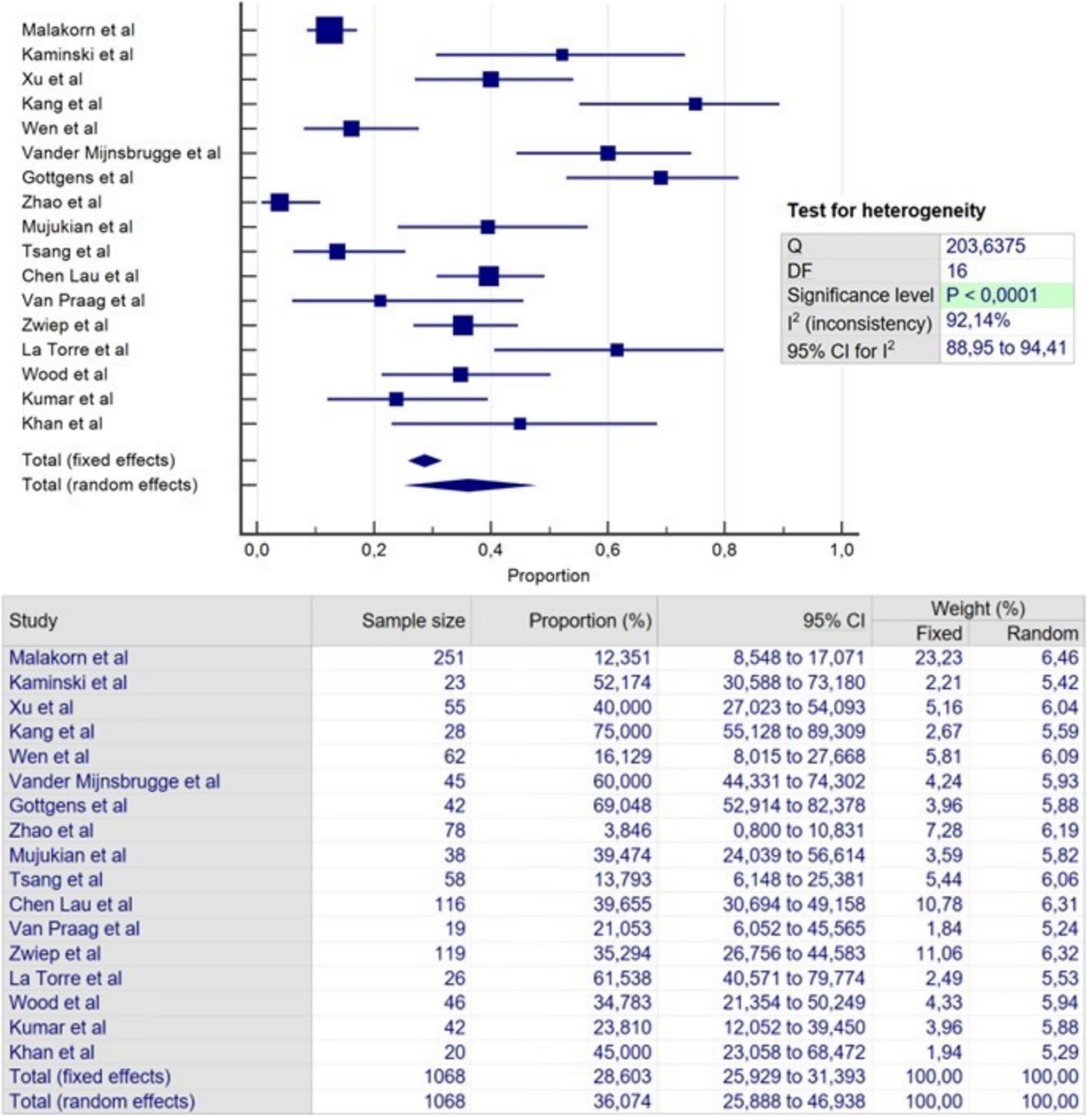
Meta-analysis of failure in LIFT

**Fig. 3:**
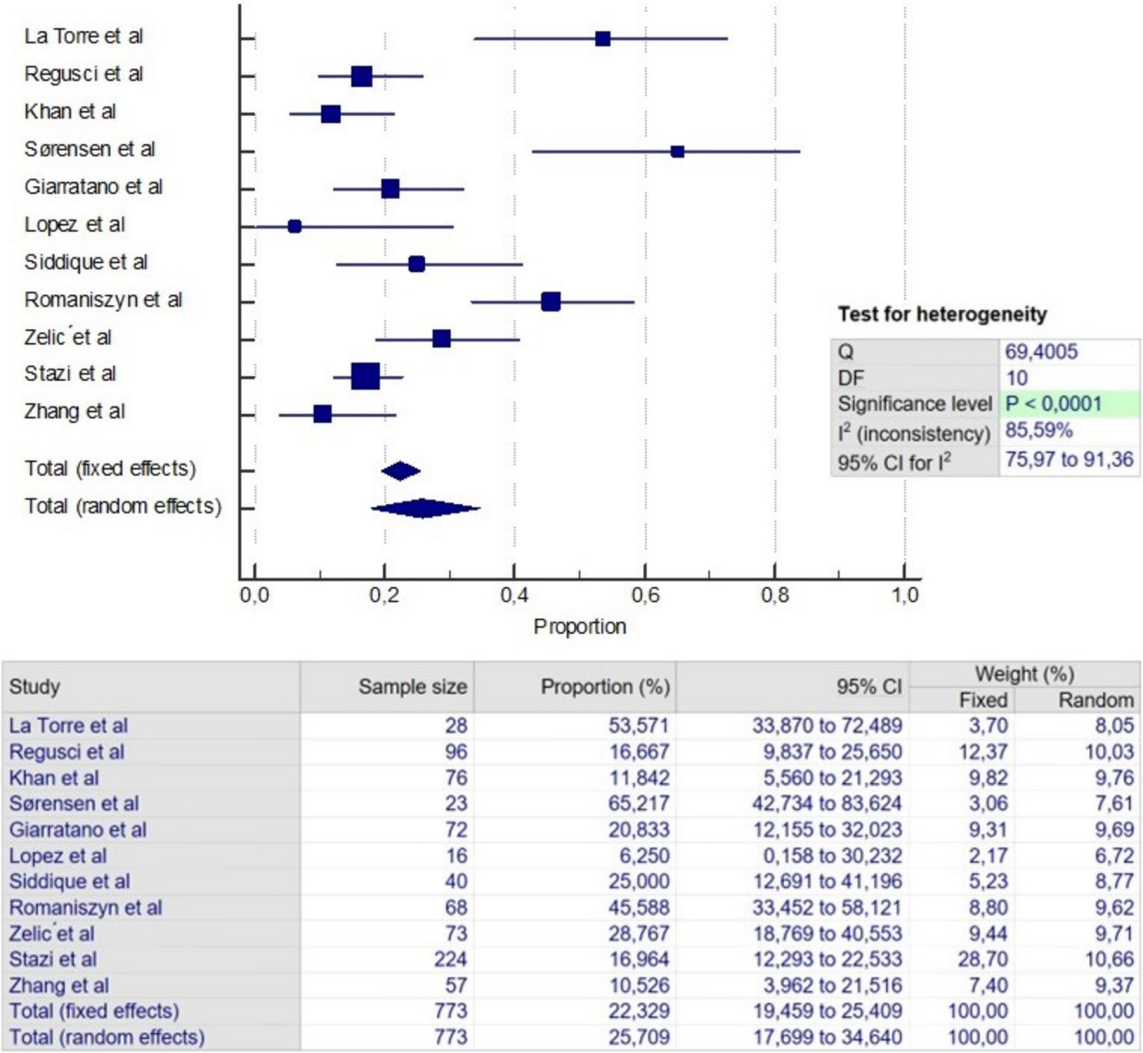
Meta-analysis of failure in VAAFT

**Fig. 4:**
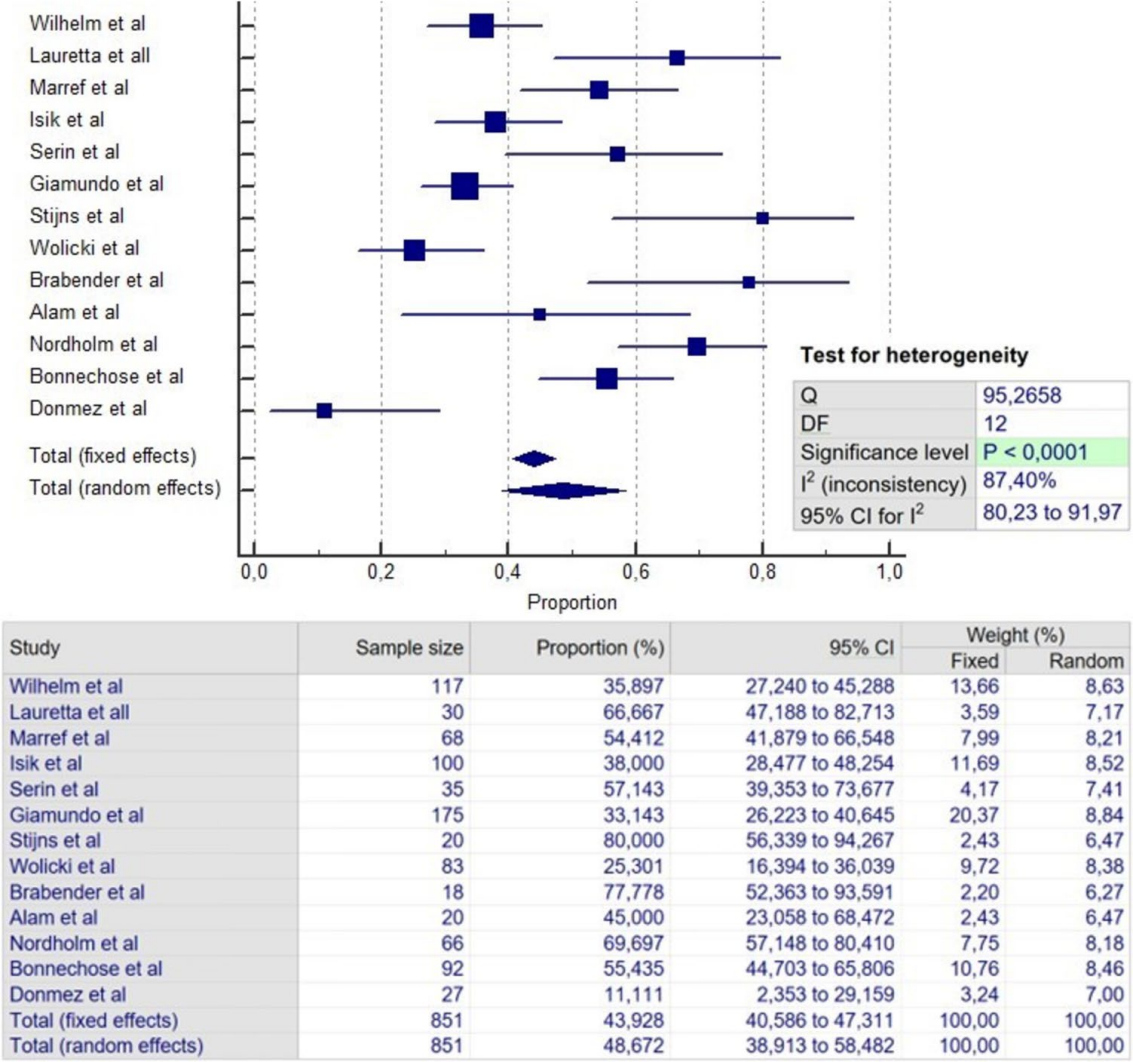
Meta-analysis of failure in FILAC

**Fig. 5:**
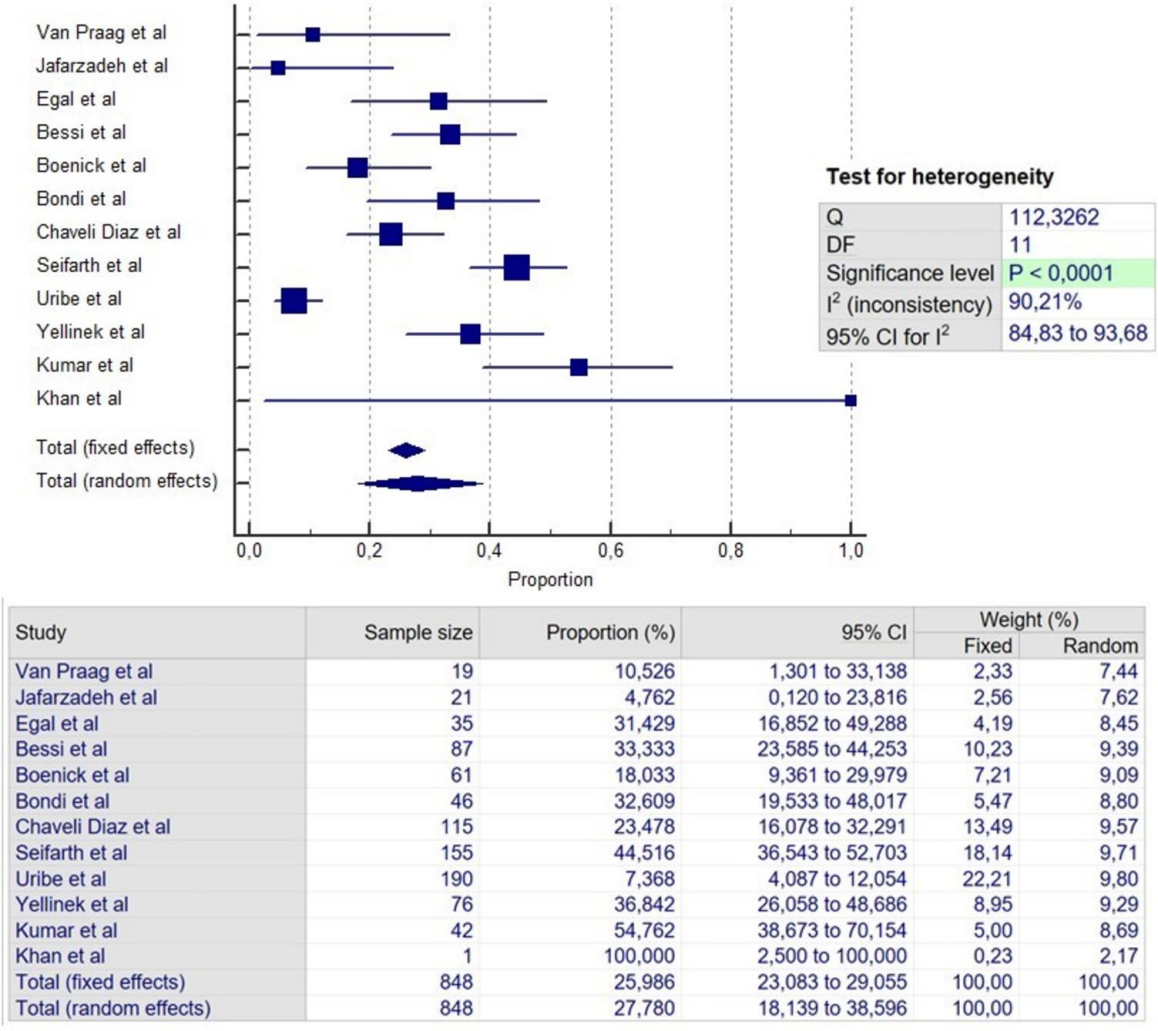
Meta-analysis of failure in EAF

All included studies provided data regarding failure. The total failure rate of the four procedures was 31.4% at an average FU of 35.6 months (Table 5). The failure rates for the four techniques were 28.6% (Fig. 2), 22.3% (Fig. 3), 43.9% (Fig. 4) and 25.9% (Fig. 5) for LIFT, VAAFT, FiLaC and EAF, respectively. The longest reported average FU was for EAF with 42.8 months, while the shortest was for studies with patients undergoing FiLaC with 31.6 months.

**Table 5:** Failure, continence disturbance and complication

	Failure				Incontinence			Minor complication			
	N studies	N patient (range)	Mean FU (range)	Failure rate (range)	N studies	N patients	Rate	N studies	N patients	Rate	Type of complications (%)
**LIFT**	17	1068	35.4 (6–80.4)	28.6% (3.8–75)	14	976	1.5%	12	806	4.3%	Wound infection (2%) Pain (1.1%) Bleeding (0.7%)
**VAAFT**	11	773 (16–224)	32.4 (6–48)	22.3% (6.2–65.2)	7	441	0%	9	710	7.2%	Perianal edema (2.4%) Bleeding (2.1%) Pain (0.7%)
**FILAC**	13	851 (18–175)	31.6 (6.3–60)	43.9% (11.1–80)	10	653	0%	11	683	10.2%	Perianal sepsis (4.2%) Pain (1.9%) Bleeding (0.3%)
**EAF**	12	848 (1–190)	42.8 (12–155)	25.9% (4.7–100)	5	232	7.3%	5	467	6.2%	Pain (6.2%) Wound infection (6.2%) Bleeding (1.2%)
**Total**	**49**	**3520**	**35,6**	**31,4%**	**34**	**2302**	**1,4%**	**34**	**2666**	**6,9%**	**Pain (1.4%)** **Wound infection (1.2%)** **Bleeding (1.1%)**

**Fig. 6:**
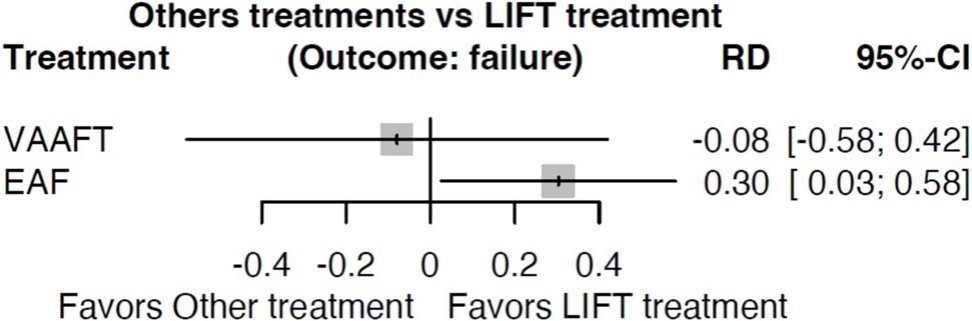
Network meta-analysis

The network meta-analysis on failure in the above four techniques showed an RD of − 0.08 (95% CI − 0.58 to 0.42) comparing LIFT vs VAAFT and a RD of 0.30 (95% CI 0.03 to 0.58) comparing LIFT vs EAF (Fig. 6).

Further analysis was done by dividing the studies into two groups based on the length of FU. The outcomes according to FU duration were as follows (< 24 vs ≥ 24 months): VAAFT, 26.1% vs 21.5, LIFT 43.2% vs 20.2%, FiLaC 56.1% vs 35.1%, EAF 36.5% vs 18.1%.

### Continence disturbance

Thirty-four studies with 2302 patients reported continence disturbance information. The total continence disturbance rate was 1.4%. No patients undergoing VAAFT or FiLaC reported worsening continence, while for LIFT the rate was 1.5% and 7.3% for EAF. Table 5 summarizes the data.

### Complications

Thirty-four studies with 2666 patients reported data on surgical complications related to the techniques. No major complications were observed, while the most common minor complication was pain (1.4%) followed by wound infection (1.2%). EAF was associated with the highest rate of pain (6.2%) and bleeding (1.2%), while FiLaC was associated with the highest overall complication rate (10.2%). Table 5 provides detailed information on complications.

### Crohn’s disease

Supplementary Table 2 refers to the failure shown in studies exclusively including patients with Crohn’s disease. The global failure rate was 39.5% at an average FU of 23.4 months. Most data (four studies with 126 patients) came from LIFT studies, with a rate of 37.3% at 25.9 months of average FU.

### Quality of studies

The overall strength of evidence is summarized in Supplementary Table 3. Low risk of bias was found for 94% of the studies, scoring between 8 and 10 for case series and between 10 and 13 for randomized trials. Two studies had medium risk with scores between 5 and 7 for case series and one randomized trial had a score of 9.

## Discussion

The results of the current meta-analysis show that FiLaC, VAAFT, LIFT and EAF have good safety profiles with acceptable failure rates, at least within the first 2 years after surgery.

LIFT showed failure rates of 28.6% at approximately 28 months of FU. Similar rates have been reported in previous studies. A meta-analysis by Hong et al. [62] reported a LIFT success rate of around 76% with a mean FU of 10 months, later confirmed in 2020 by another meta-analysis [63]. The latter study reports a 9.6% fistula persistence rate and 12.4% recurrence rate. The current review updates those figures and provides outcomes at longer FU intervals.

Compared with LIFT, VAAFT was found to be associated with lower failure rates (22.3%) at similar FU (average 32.4 months) but without a statistical difference (RD − 0.08 95% CI − 0.58 to 0.42). A recent meta-analysis [64] on VAAFT reported a recurrence rate of 17.1 within 9 months, and the current study suggests that higher rates can be expected within 2 years.

A study published in 2020 by Regusci et al. [30] reported a success rate of 83.3% at 36-month FU, while a recent randomized trial allocating patients to either VAAFT or fistulectomy for high cryptoglandular fistulas reported a recurrence rate of 65% vs 27% at 6-month FU [32].

In contrast, the present study shows a statistically significant difference when comparing LIFT and EAF, with an increased risk of failure for EAF (RD 0.30 (CI95% 0.03–0.58), despite reporting a failure rate of 25.8%.

According to the current metanalysis, the highest failure rates occurred after FiLaC (43.9%), with a mean FU of of 31.6 months, and similar figures have been previously reported [65, 66].

Few studies focused on Crohn’s disease. The current study found an increased failure rate after EAF, FiLaC and LIFT in this population, but data were obtained from a single study for FiLaC with short FU (7 months). No included studies report results on VAAFT, while more data are available concerning LIFT, which shows a safe profile for the technique and an acceptable failure rate (approximately 37%). The most recent guidelines of the European Crohn’s and Colitis Organisation (ECCO) suggested that LIFT is an option in the treatment of complex anal fistulas in selected Crohn’s disease patients [67]. It is useful to specify that in Crohn’s disease the failure rate may be affected by numerous factors such as whether the patient is in biological therapy and whether the procedure was performed during or after induction therapy, because all of this can have a significant impact on healing rates. Unfortunately, it is difficult to extract this information from the studies; therefore, this finding should be considered with caution.

In terms of continence disturbance, both VAAFT and FiLaC were not associated with continence disturbance, whereas LIFT showed a 1.5% continence disturbance rate among a much higher number of included patients (976 vs 441 undergoing VAAFT and 653 undergoing FiLaC). EAF, on the other hand, showed the highest rate of continence disorders with 7.3% but in a significantly smaller patient population (232 patients). Very few studies in the literature report on patients with worsening continence post-LIFT technique [19, 24]. A recent pooled meta-analysis showed a post EAF incontinence rate of 7.8%, in agreement with the data shown by the present work [68].

One should also consider that in some studies this could have not been routinely or objectively investigated. The included procedures are considered sphincter-saving approaches [1], but this definition should be accepted with caution. Both LIFT and VAAFT usually require an internal flap to close the site of the previous internal orifice, which encompass internal sphincter fibers, so that this minimal muscle damage may reveal subclinical fecal incontinence. Furthermore, the retractor used is not systematically reported and may represent a potential cause of subclinical sphincter damage [69].

The four techniques have been shown to have a safe profile with no major complications. Most common complications are bleeding, postoperative pain and wound infection. Irrespective of the failure rates, one should consider that these techniques, in particular VAAFT and FiLaC, have the advantage of minimal morbidity, which makes it possible to repeat the procedures in case of initial failure. Even if acknowledging the importance of avoiding morbidity, it is also important to emphasize that failure could result in a relevant psychophysical trauma for the patient, and it may represent a challenge for surgeons. Furthermore, even if minimally invasive, previous surgery could result in scar formation that could reduce the success of a more invasive but resolving technique.

Some have reported extremely low VAAFT-related cure rates, 22% for cryptoglandular and 27% for Crohn's disease fistulas [70]. However, up to 40% of the unhealed fistulas showed significant improvement in symptoms, which would suggest that the technique may be useful in controlling symptoms even if not reaching a fistula cure. This concept has been recently popularized by Adegbola et al. for complex Crohn’s disease fistulas refractory to treatment [71].

Concerning LIFT, it is important to consider that a failure of this procedure could result in a “simpler” fistula, making subsequent procedures easier. In the current analysis, persistence or recurrence of the fistula was considered a failure itself, but one should also take this concept into account when considering adopting one treatment against the other.

### Strengths and limitations

This meta-analysis pooled data on four relatively novel sphincter-preserving procedures and included a substantial number of studies. It produced information that might be useful when deciding which technique might suit each patient best and providing them with realistic expectations. However, most of these articles are retrospective and observational with heterogeneous FU, often < 1 year. High heterogeneity of data was observed. In addition, the frequent absence of comparison groups and also the different methods for continence assessment are further limitations of the current study.

Another consideration, which might skew the data, is that VAAFT and FiLaC are procedures requiring special instrumentation, with specific costs, with potential conflicts of interest. In terms of cost, LIFT and EAF are probably the least expensive, since they do not require any special instrumentation, assuming they are performed on the day of surgery. Cost-effectiveness data concerning these procedures are lacking.

In addition, intraoperative data that may affect postoperative outcomes, such as the retractor or type of flap used, the closure or non-closure of the internal opening and the type of closure, are not systematically reported, which contributed to generating a further relevant bias as it prevented detailed sub-analyses. Therefore, it was not possible to unequivocally demonstrate the superiority of one technique over another.

Lastly, several minimally invasive approaches have been proposed in recent years. The aim of this meta-analysis was to compare the four most widely used minimally invasive techniques, but it is useful to remember that some of the other techniques have shown encouraging results and have sometimes been used in combination with those investigated in this article, sometimes improving the results of one technique alone [22, 72].

## Conclusion

FiLaC, VAAFT, LIFT and EAF may represent a safe option in the treatment of anal fistula. Long-term FU data are lacking, and many studies are observational with all inherent biases; the data available show no associated major complications with an acceptable failure rate and impact on continence. Figures on recurrence seem to increase with longer FU. The data of this meta-analysis showed a lower failure rate for VAAFT, but readers are advised to read findings carefully, given the uncertain statistical significance and many variants and heterogeneities in available studies. Homogeneous, clear and long-term FU studies are necessary to obtain more robust data.

## Supplementary Information

Below is the link to the electronic supplementary material.

Supplementary Table 1. Detailed search strategy (DOCX 13 KB)

Supplementary Table 2. Failure in Crohn's disease (DOCX 14 KB)

Supplementary Table 3. Bias assessment for included studies according to the Joanna Briggs Institute's (JBI) critical appraisal checklist (DOCX 35 KB)

## Data Availability

No datasets were generated or analyzed during the current study.
