# Supplementary material for: Failure rates and complications of four sphincter-sparing techniques for the treatment of fistula-in-ano: a systematic review and network meta-analysis
Source: Tech Coloproctol. 2025 May 20;29(1):116. doi: 10.1007/s10151-025-03152-0 (PMC12092498; doi:10.1007/s10151-025-03152-0)
Supplement: Supplementary file 1 — Supplementary Table 1. Detailed search strategy (DOCX 13 KB) [file 10151_2025_3152_MOESM1_ESM.docx]

| **Database** | **Time span** | **Search strategy** | **Records identified** |
| --- | --- | --- | --- |
| MEDLINE (Pubmed) | March 15th 2023 | (ligation of intersphincteric fistula tract[Title/Abstract]) OR (video assisted anal fistula treatment[Title/Abstract]) OR (fistula laser closure[Title/Abstract]) OR (endorectal flap[Title/Abstract]) OR (endoanal flap[Title/Abstract]) | 173 |
| Embase | March 15th 2023 | 'ligation of intersphincteric fistula tract':ab,ti OR 'video assisted anal fistula treatment':ab,ti OR 'fistula laser closure':ab,ti OR ‘endoanal flap’:ab,ti OR ‘endorectal flap’:ab,ti | 352 |
