# Supplementary material for: Failure rates and complications of four sphincter-sparing techniques for the treatment of fistula-in-ano: a systematic review and network meta-analysis
Source: Tech Coloproctol. 2025 May 20;29(1):116. doi: 10.1007/s10151-025-03152-0 (PMC12092498; doi:10.1007/s10151-025-03152-0)
Supplement: Supplementary file 2 — Supplementary Table 2. Failure in Crohn's disease (DOCX 14 KB) [file 10151_2025_3152_MOESM2_ESM.docx]

|  | N studies CD | N patients CD | Failure rate in CD | Mean FU CD |
| --- | --- | --- | --- | --- |
| LIFT | 4 | 126 | 37,3% | 25,9 months |
| VAAFT | 0 | 0 | - | - |
| FILAC | 1 | 20 | 45% | 7 months |
| EAF | 2 | 74 | 41,9% | 23,5 months |
| Total | **7** | **220** | **39,5%** | **23.4 months** |
