# Supplementary material for: Failure rates and complications of four sphincter-sparing techniques for the treatment of fistula-in-ano: a systematic review and network meta-analysis
Source: Tech Coloproctol. 2025 May 20;29(1):116. doi: 10.1007/s10151-025-03152-0 (PMC12092498; doi:10.1007/s10151-025-03152-0)
Supplement: Supplementary file 3 — Supplementary Table 3. Bias assessment for included studies according to the Joanna Briggs Institute's (JBI) critical appraisal checklist (DOCX 35 KB) [file 10151_2025_3152_MOESM3_ESM.docx]

| **CASE SERIES** | | | | | | | | | | | |
| --- | --- | --- | --- | --- | --- | --- | --- | --- | --- | --- | --- |
| **Articles** | **Q1** | **Q2** | **Q3** | **Q4** | **Q5** | **Q6** | **Q7** | **Q8** | **Q9** | **Q10** | **SCORE** |
| La Torre et al | YES | YES | YES | YES | YES | NO | YES | YES | NO | YES | **8/10** |
| Regusci et al | YES | YES | YES | YES | YES | YES | YES | YES | YES | YES | **10/10** |
| Khan et al | YES | YES | YES | YES | YES | YES | YES | YES | NO | YES | **9/10** |
| Giarratano et al | YES | YES | YES | YES | UN | YES | YES | YES | NO | YES | **8/10** |
| Lopez et al | YES | YES | NO | YES | YES | YES | YES | YES | NO | YES | **8/10** |
| Romaniszyn et al | YES | YES | YES | YES | YES | YES | NO | YES | NO | YES | **8/10** |
| Zelić et al | YES | YES | YES | YES | UN | YES | UN | YES | NO | YES | **7/10** |
| Stazi et al | YES | YES | NO | YES | YES | YES | YES | YES | YES | YES | **9/10** |
| Zhang et al | YES | YES | NO | UN | UN | YES | YES | YES | YES | YES | **7/10** |
| Wilhelm et al | YES | YES | YES | UN | UN | YES | YES | YES | YES | YES | **8/10** |
| Lauretta et al | YES | YES | YES | YES | UN | YES | YES | YES | YES | YES | **9/10** |
| Marref et al | YES | YES | NO | YES | YES | YES | YES | YES | YES | YES | **9/10** |
| Isik et al | YES | YES | YES | YES | YES | YES | YES | YES | YES | YES | **10/10** |
| Serin et al | YES | YES | YES | YES | UN | YES | YES | YES | YES | YES | **9/10** |
| Giamundo et al | YES | YES | YES | YES | UN | YES | YES | YES | YES | YES | **9/10** |
| Stijns et al | YES | YES | YES | NO | NO | YES | YES | YES | YES | YES | **8/10** |
| Wolicki et al | YES | YES | YES | YES | UN | YES | YES | YES | YES | YES | **9/10** |
| Brabender et al | YES | YES | YES | YES | YES | YES | YES | YES | UN | YES | **9/10** |
| Alam et al | YES | YES | NO | YES | YES | YES | YES | YES | YES | YES | **9/10** |
| Nordholm-Carstensen et al | YES | YES | YES | YES | YES | YES | YES | YES | YES | YES | **10/10** |
| Bonnechose et al | YSE | YES | YES | YES | UN | YES | YES | YES | YES | YES | **9/10** |
| Donmez et al | YES | YES | YES | YES | UN | YES | YES | YES | NO | YES | **8/10** |
| Malakorn et al | YES | YES | YES | YES | YES | YES | YES | YES | YES | YES | **10/10** |
| Kaminski et al | YES | YES | YES | YES | UN | YES | YES | YES | YES | YES | **9/10** |
| Xu et al | YES | YES | YES | YES | YES | YES | YES | YES | UN | YES | **9/10** |
| Kang et al | YES | YES | YES | YES | UN | YES | YES | YES | UN | YES | **8/10** |
| Wen et al | YES | YES | YES | YES | UN | YES | YES | YES | NO | YES | **8/10** |
| Vander Mijnsbrugge et al | YES | YES | YES | YES | YES | YES | YES | YES | NO | YES | **9/10** |
| Gottgens et al | YES | YES | YES | YES | YES | YES | UN | YES | UN | YES | **8/10** |
| Zhao et al | YES | YES | YES | YES | YES | YES | NO | YES | NO | YES | **8/10** |
| Mujukian et al | YES | YES | YES | YES | YES | YES | YES | YES | YES | YES | **10/10** |
| Tsang et al | YES | YES | UN | YES | YES | YES | YES | YES | UN | YES | **8/10** |
| Chen Lau et al | YES | YES | YES | YES | YES | YES | YES | YES | YES | YES | **10/10** |
| Van Praag et al | YES | YES | YES | YES | YES | YES | YES | YES | YES | YES | **10/10** |
| Zwiep et al | YES | YES | YES | YES | YES | YES | YES | YES | NO | YES | **9/10** |
| Wood et al | YES | YES | UN | YES | YES | YES | YES | YES | YES | YES | **9/10** |
| Khan et al | YES | YES | YES | YES | YES | YES | YES | YES | NO | YES | **9/10** |
| Egal et al | YES | YES | UN | YES | YES | YES | YES | YES | YES | YES | **9/10** |
| Bessi et al | YES | YES | UN | YES | UN | YES | YES | YES | YES | YES | **8/10** |
| Boenicke et al | YES | YES | YES | YES | YES | YES | YES | YES | YES | YES | **10/10** |
| Chaveli Diaz | YES | YES | YES | YES | YES | NO | YES | YES | NO | YES | **8/10** |
| Seifarth et al | YES | YES | UN | YES | YES | YES | YES | YES | YES | YES | **9/10** |
| Uribe et al | YES | YES | UN | YES | YES | YES | YES | YES | YES | YES | **9/10** |
| Yellinek et al | YES | YES | UN | YES | YES | YES | YES | YES | YES | YES | **9/10** |

| **RANDOMIZED TRIAL** | | | | | | | | | | | | | | |
| --- | --- | --- | --- | --- | --- | --- | --- | --- | --- | --- | --- | --- | --- | --- |
| **Articles** | **Q1** | **Q2** | **Q3** | **Q4** | **Q5** | **Q6** | **Q7** | **Q8** | **Q9** | **Q10** | **Q11** | **Q12** | **Q13** | **SCORE** |
| Sørensen et al | YES | YES | YES | NO | NO | UN | YES | YES | YES | YES | YES | YES | YES | **10/13** |
| Siddique et al | YES | YES | YES | NO | NO | UN | YES | YES | YES | YES | YES | YES | YES | **10/13** |
| Kumar et al | YES | YES | YES | NO | NO | UN | YES | YES | YES | YES | YES | YES | YES | **10/13** |
| Jafarzadeh et al | UN | YES | YES | NO | NO | UN | YES | YES | YES | YES | YES | YES | YES | **9/13** |
| Bondi et al | YES | YES | YES | NO | NO | UN | YES | YES | YES | YES | YES | YES | YES | **10/13** |

| **CASE SERIES** | |
| --- | --- |
| **Q1** | Were there clear criteria for inclusion in the case series? |
| **Q2** | Was the condition measured in a standard, reliable way for all participants included in the case series? |
| **Q3** | Were valid methods used for identification of the condition for all participants included in the case series? |
| **Q4** | Did the case series have consecutive inclusion of participants? |
| **Q5** | Did the case series have complete inclusion of participants? |
| **Q6** | Was there clear reporting of the demographics of the participants in the study? |
| **Q7** | Was there clear reporting of clinical information of the participants? |
| **Q8** | Were the outcomes or follow up results of cases clearly reported? |
| **Q9** | Was there clear reporting of the presenting site(s)/clinic(s) demographic information? |
| **Q10** | Was statistical analysis appropriate? |

| **RANDOMIZED TRIAL** | |
| --- | --- |
| **Q1** | Was true randomization used for assignment of participants to treatment groups? |
| **Q2** | Was allocation to treatment groups concealed? |
| **Q3** | Were treatment groups similar at the baseline? |
| **Q4** | Were participants blind to treatment assignment? |
| **Q5** | Were those delivering treatment blind to treatment assignment? |
| **Q6** | Were outcomes assessors blind to treatment assignment? |
| **Q7** | Were treatment groups treated identically other than the intervention of interest? |
| **Q8** | Was follow up complete and if not, were differences between groups in terms of their follow up adequately described and analyzed? |
| **Q9** | Were participants analyzed in the groups to which they were randomized? |
| **Q10** | Were outcomes measured in the same way for treatment groups? |
| **Q11** | Were outcomes measured in a reliable way? |
| **Q12** | Was appropriate statistical analysis used? |
| **Q13** | Was the trial design appropriate, and any deviations from the standard RCT design (individual randomization, parallel groups) accounted for in the conduct and analysis of the trial? |
